# Discovery and identification of medium‐chain fatty acid responsive promoters in *Saccharomyces cerevisiae*

**DOI:** 10.1002/elsc.201900093

**Published:** 2020-01-21

**Authors:** Li Han, Danya Han, Lei Li, Shen Huang, Peixin He, Qinhong Wang

**Affiliations:** ^1^ Henan Collaborative Innovation Center for Food Production and Safety School of Food and Bioengineering Zhengzhou University of Light Industry Zhengzhou P. R. China; ^2^ Henan Key Laboratory of Cold Chain Food Quality and Safety Control Zhengzhou P. R. China; ^3^ CAS Key Laboratory of Systems Microbial Biotechnology Tianjin Institute of Industrial Biotechnology Chinese Academy of Sciences (CAS) Tianjin P. R. China

**Keywords:** dose response, medium‐chain fatty acid, promoter, *Saccharomyces cerevisiae*, transcriptome

## Abstract

Medium‐chain fatty acids (MCFAs) and their derivatives are important chemicals that can be used in lubricants, detergents, and cosmetics. MCFAs can be produced in several microbes, although production is not high. Dynamic regulation by synthetic biology is a good method of improving production of chemicals that avoids toxic intermediates, but chemical‐responsive promoters are required. Several MCFA sensors or promoters have been reported in *Saccharomyces cerevisiae*. In this study, by using transcriptomic analysis of *S. cerevisiae* exposed to fatty acids with 6‐, 12‐, and 16‐carbon chains, we identified 58 candidate genes that may be responsive to MCFAs. Using a fluorescence‐based screening method, we identified MCFA‐responsive promoters, four that upregulated gene expression, and three that downregulated gene expression. Dose–response analysis revealed that some of the promoters were sensitive to fatty acid concentrations as low as 0.02–0.06 mM. The MCFA‐responsive promoters reported in this study could be used in dynamic regulation of fatty acids and fatty acid‐derived products in *S. cerevisiae*.

**Abbreviation**
MCFAs: medium‐chain fatty acids

## 1 INTRODUCTION

With the development of metabolic engineering and synthetic biology, more and more compounds can be produced in microorganisms, including natural terpenoid chemicals, antibiotics, biofuels, pharmaceuticals, and other popular chemical products 1, 2. To avoid metabolic imbalance caused by overproduction of toxic enzymes or intermediates that lead to growth retardation and yield reduction, dynamic regulation with a sensor was proposed and achieved 3, 4. Dynamic regulation exists in natural organisms, which use transcriptional and translational control, while synthetic dynamic control always consists of a biosensor measuring key intermediates and cognate regulators 5, 6. By using dynamic regulation strategies, production of several chemicals, including farnesyl pyrophosphate (FPP) 4, 7, malonyl‐CoA 8, 9, and fatty acids 6, has been improved in engineered microorganisms. Biosensors can also be used in high‐throughput screening to sense and respond to the desired product 10, 11.

A broad range of intermediate metabolites, exogenous stimuli (inducers and light), environmental signals (pH, oxygen, and temperature), and molecules that reflect cellular growth status can be sensed 5, 12, 13. Intracellular metabolite‐responsive sensors can sometimes tune gene expression and intermediate accumulation to improve metabolic balance and productivity. So far, a few intracellular metabolic sensors have been applied in synthetic biology, and most such biosensors have been designed based on existing transcription factors and riboswitches 14, 15, 16, 17. Some new biosensors were also developed by using rational design or random mutagenesis approaches to alter the effector specificities of reported genetic components 12, 18, 19. The development of dynamic regulation by using various sensors in *Escherichia coli* is much faster than that in other species such as *Saccharomyces cerevisiae*, *Corynebacterium glutamicum*, and *Aspergillus niger*. However, there are several advantages of production of valuable chemicals in the latter microorganisms because of their different characteristics compared with *E. coli*. Thus, it is necessary to find more sensors that can be applied in dynamic regulation in more microbes.

Fatty acids and their derivatives including fatty alcohols, triacylglycerols, fatty acid ethyl esters, and alkanes have been synthesized in microbes such as *E. coli*, *S. cerevisiae*, and oleaginous yeast 20. Compared with long‐chain fatty acids (LCFAs), medium‐chain fatty acids (MCFAs; chains of six to 12 carbon atoms [C6–C12]) have some advantages, such as improved biofuel quality and suitability as substitutes for fossil fuels. Moreover, MCFAs have been used in lubricants, detergents, and cosmetics 21. Some work has been done to produce MCFAs in microbes 22. Several sensors have been used to regulate and improve fatty acid production in *E. coli*, such as FadR[6] and FapR 8, 23. The bacterial sensors FadR 24 and malonyl‐CoA sensor FapR 25, 26 have also been successfully expressed and used in *S. cerevisiae*. Further, a G‐protein‐coupled receptor from mammals has been used to detect even‐chain C8–C12 fatty acids in *S. cerevisiae* 27. Recently, the endogenous short‐ and medium‐chain fatty acid promoter sensor pPDR12 of *S. cerevisiae* has been reported and showed its highest sensitivity toward C6 28. However, much more work needs to be done to study MCFA‐responsive promoters in *S. cerevisiae*.

Promoters are important elements in synthetic pathway construction and can be designed to apply dynamic control. For example, the sucrose‐inducible SUC2 promoter was applied in regulation of gene expression by using RNA interference 29, a low‐pH‐inducible promoter pGAS can promote gene expression at pH 2.0 30, and with an ergosterol‐responsive promoter, metabolic flux could be diverted from the production of sterols to the end product amorphadiene 31. High‐throughput genomics technology can help in the search for promoters responsive to stress stimulus 30, 32, 33, 34, and, by this method, an FPP‐responsive promoter was first found and then used to improve amorphadiene production by dynamic regulation in *E. coli* 4. Recently, a butanol‐responsive promoter was discovered in *S. cerevisiae* by using transcriptomic analysis 35.

Here, to find endogenous promoters responsive to MCFAs, we used transcriptomics to analyze differential gene transcript levels in *S. cerevisiae* treated with C6, C12 and C16 fatty acids. Then, the promoters of candidate genes were evaluated for their response to C6 and C12 MCFAs. Positively responsive promoters (including those that upregulated and downregulated gene expression) were chosen for analysis of their response characteristics to fatty acids (chain length and concentration). The promoters we identified may be used in dynamic regulation of synthesis of fatty acids and derived chemicals in *S. cerevisiae*.

**PRACTICAL APPLICATION**

In the present study, several promoters from *Saccharomyces cerevisiae* that can respond to medium‐chain fatty acids were identified by transcriptomic analysis. pTDH1 and pPHO3 upregulated gene expression in response to fatty acids (as did pPDR12 that has been reported previously). pHXT7 downregulated gene expression in response to fatty acids. Tests of response to different carbon‐chain length fatty acids and dose–response experiments indicated that the three promoters could respond to some fatty acids in concentrations as low as 0.02–0.06 mM. This study presents a method for finding chemical‐responsive promoters, and the fatty acid‐responsive promoters identified here have potential for use in dynamic regulation of fatty acid‐derived products in *S. cerevisiae*.

## 2 MATERIALS AND METHODS

### 2.1 Strains

*S. cerevisiae* strain BY4741 was used as the host strain in this study (Table 1). The method used for yeast transformation was the standard LiAc/SS carrier DNA/PEG method 36. Plasmid construction was performed using *E. coli* strain DH5α.

**Table 1:** Plasmids and strains used in this article

Name	Genotype	Source
Strains		
BY4741	Matα; his3Δ1; leu2Δ0; met15Δ0; ura3Δ0	Euroscarf
Plasmids		
pET30a‐eGFP	pT7‐eGFP, f1 origin, Kan	Our laboratory
pRS316	None gene, CEN6 replicon, *URA3*	Our laboratory
pRS316‐GPD‐eGFP	pGPD1‐eGFP, CEN6 replicon, *URA3*	This study
pLeu2‐GPD‐mCherry	pGPD1‐mcherry, 2‐micron replicon, *LEU2*	Our laboratory
pRS316‐GPD‐mCherry	pGPD1‐mcherry, CEN6 replicon, *URA3*	This study
pLeu2‐Empty‐mCherry	pEmpty‐mcherry, 2‐micron replicon, *LEU2*	This study

### 2.2 Media and growth conditions

All yeast and bacterial strains were stored in 30% glycerol at −80°C. *E. coli* was cultured in Luria–Bertani medium at 37°C with shaking at 200 rpm.

For transcriptome analysis, *S. cerevisiae* strains were cultivated in YPD medium (10 g/L yeast extract, 20 g/L peptone, and 20 g/L glucose) at 30°C with agitation at 200 rpm in baffled flasks. When cells reached early log phase, 1 mM caproic acid (C6), 1 mM lauric acid (C12), or 1 mM hexadecanoic acid (C16) was added to the medium; 600 µL cosolvent (isopropanol:Triton X‐100, 3:2 v:v) was added to 30 mL YPD medium with each fatty acid. In controls, 600 µL cosolvent without fatty acid were added to the medium. After about 6 h, each sample was collected for analysis.

To test for response to fatty acids, a fresh colony of *S. cerevisiae* grown at 30°C on synthetic dextrose (SD) solid medium (6.7 g/L yeast nitrogen base [Sigma], 20 g/L glucose, yeast synthetic drop‐out medium supplements without histidine, leucine, tryptophan, and uracil [Sigma], 20 g/L agar, and a mixture of appropriate amino acids) was inoculated into 5 mL SD medium with initial OD_600_ of about 0.05. Caproic acid (C6) or lauric acid (C12) was added to 1 mM. In controls, no fatty acid was added. After about 16 h, cells were collected for the measurement of fluorescence intensity.

Minimal medium contained 7.5 g/L (NH_4_)_2_SO_4_, 14.4 g/L KH_2_PO_4_, 0.5 g/L MgSO_4_∙7H_2_O, 20 g/L glucose, trace metal and vitamin solutions, and appropriate amino acid supplements as needed 37.

### 2.3 Transcriptome analysis

Samples were collected and then sent to BGI Co., Ltd. for transcriptome sequencing using Illumina HiSeq 4000 Technology. The sequenced reads were mapped to the reference genome of *S. cerevisiae* using Bowtie2 38 and the expression levels in FPKM (Fragments Per Kilobase of transcript per Million mapped reads) were calculated using RSEM 39. Analysis of differential expression of genes was performed using PossionDis (fold‐change ≥ 2.00 and false discovery rate ≤ 0.001) 40. The log ratios were hierarchically clustered using Genesis 41. Subsequently, Gene Ontology (GO) analysis was performed by using the DAVID Functional Annotation Tool 42 with the functional annotation chart produced with GOTERM_BP_DIRECT.

### 2.4 Plasmid construction

Plasmids pRS316 and pLeu2‐GPD‐mCherry were from our laboratory 43 (Table 1). The enhanced green fluorescent protein (eGFP)‐encoding gene was amplified with primers eGFP (Table S1) from pET30a‐eGFP (from our laboratory) and then the fragment was cut with *Nco*I and *Hin*dIII. The fragment was ligated into pLeu2‐GPD‐mCherry cut with the same enzymes to construct pGPD1‐eGFP‐TCYC1. The expression cassette of pGPD1‐eGFP‐TCYC1 was removed using restriction enzymes *Sac*I and *Kpn*I and ligated into pRS316 cut with the same enzymes to construct pRS316‐GPD‐eGFP. The promoters of candidate genes (enough to cover all of the transcription factor binding sites, i.e., about 1.5 kb) were amplified with corresponding primers (Table S1) based on the genome database of *S. cerevisiae*. Then each amplified fragment was cut by *Sac*I (some with *Spe*I) and *Bam*HI and ligated into pLeu2‐GPD‐mCherry to replace promoter GPD1. Plasmid pLeu2‐Empty‐mCherry was constructed by removing the GPD1 promoter. Selected promoters and promoter GPD1 controlling expression of mCherry were also respectively put into single‐copy plasmid pRS316. All primers were synthesized by Sheng Gong Corporation, Shanghai, and restriction enzymes were purchased from New England Biolabs.

## 3 RESULTS AND DISCUSSION

### 3.1 Screening of possible MCFA‐responsive promoters by transcriptomic analysis of *S. cerevisiae*

Previously, it was demonstrated that there were two different resistance mechanisms in *S. cerevisiae* to octanoic acid (C8) and decanoic acid (C10), the response to which shared many genes with an oxidative stress response 44, 45. To find promoters responsive to MCFAs, we used a transcriptome sequencing method to analyze differential transcript levels in *S. cerevisiae* exposed to different carbon chain‐length fatty acids—caproic acid (C6), dodecanoic acid (C12), and hexadecanoic acid (C16). Each fatty acid (1 mM) was respectively added to cells in the initial exponential growth stage, and then samples were collected later in the exponential growth period for transcriptome analysis. The chosen concentration of fatty acids had little impact on cell growth, but was sufficient for induction of the stress resistance response 44.

In the transcriptome analysis, fewer genes were differentially expressed upon C16 treatment compared with C12 and, especially, C6 treatment (Figure S1A). The main fatty acid products during the fermentation of *S. cerevisiae* are C16 and C18, presumably explaining why treatment with hexadecanoic acid (C16) was less toxic to the cells than treatment with the C6 and C12 fatty acids. GO analysis revealed that genes from many metabolic processes were influenced by C6, C12, and C16, and many differentially expressed genes on C6 treatment were involved in transport and oxidation/reduction processes (Figure S1B and S1C).

**Figure 1:**
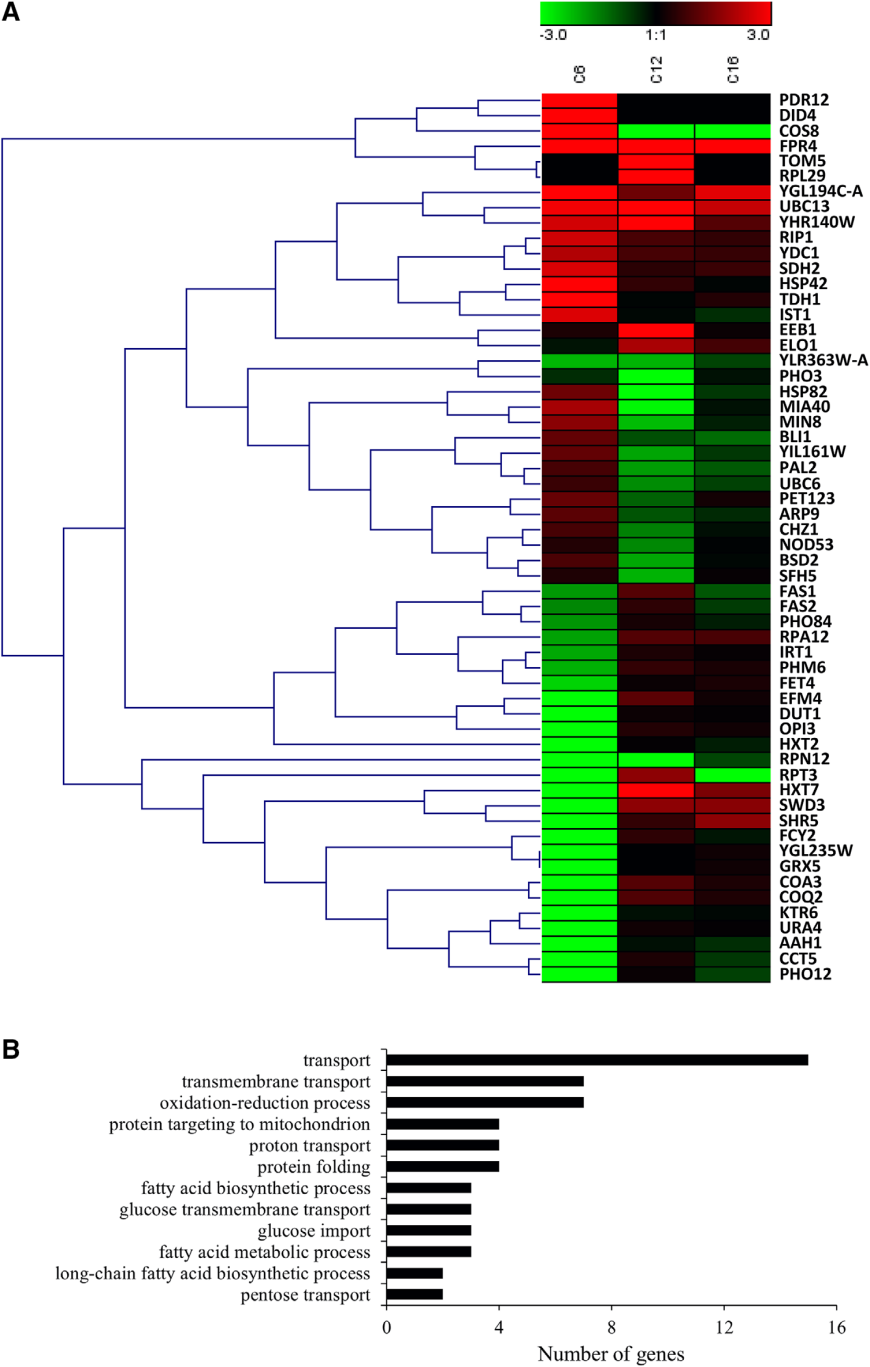
Hierarchical clustering of expression of 58 selected genes (log_2_ ratio FAs/Control) from *Saccharomyces cerevisiae* exposed to different chain‐length fatty acids (C6, C12, and C16; A) and Gene Ontology analysis of these genes (B)

The goal of the present work was to find fatty acid‐responsive promoters in *S. cerevisiae*, especially those responsive to MCFAs. First, we divided the transcriptomic data into two groups—genes that were upregulated, and those that were downregulated (we wanted to find promoters that can promote or inhibit gene expression in response to fatty acids). In the upregulation group, genes with expression level FPKM ≥20 that were specifically upregulated by C6 or C12 fatty acid (log_2_ ratio ≥1) were selected first. Then, genes (FPKM ≥20) obviously and simultaneously upregulated by C6 and C12 (log_2_ ratio ≥1) but not LCFA (C16) were selected to help search for promoters specifically responsive to MCFAs. Finally, several genes (FPKM ≥20) with high log_2_‐fold change value but simultaneously upregulated by C6, C12, and C16 were selected to see whether the promoters of these genes respond nonspecifically to MCFAs. In the downregulation group, the screening strategy was similar to that for the upregulation group, but an expression level of FPKM ≥20 was required in the control sample to eliminate possible read errors, and an absolute value of log_2_ ratio ≥1 for differential expression was needed. In addition, some genes that were not expressed when C6 was added to the medium (FPKM ≥20 in the control sample) with an absolute value of log_2_ ratio > 10 were chosen for further analysis. There were similar genes on C12 addition, but the FPKM value in the control sample was < 20, so there was no selection of this type of data (Table S2). The screened genes of the promoter were then hierarchically clustered, and GO analysis was performed. We found that most of the selected genes were involved in transportation or metabolic processes such as the fatty acid biosynthesis pathway, and several genes were involved in oxidation/reduction processes (Figure 1).

### 3.2 Identification and confirmation of fatty acid‐responsive promoters

Quantitative RT‐PCR experiments were performed on nine randomly chosen genes from among the 58 selected genes (Figure 1). The expression changes determined by transcriptomics were validated for all of them, so we concluded that the transcriptomic analysis was reliable.

To determine whether the promoters of the 58 selected genes (Figure 1) responded to MCFAs, reporter assays were performed. Each promoter (from 1 bp to around 1500 bp upstream of the translation start codon) was ligated before a gene encoding mCherry red fluorescent protein. As the positive control, constitutive promoter GPD1 was ligated before mCherry. Furthermore, eGFP was also ligated after the GPD1 promoter in plasmid pRS316‐GPD‐eGFP and then co‐transformed with plasmid pLeu2‐promoter‐mCherry containing the selected (test) promoter into *S. cerevisiae* strain BY4741. Then, the ratio of mCherry/eGFP fluorescence was determined to evaluate the test promoter.

To verify that this method could be used to screen promoters, we took 24 samples of strains that carried plasmids pRS316‐GPD‐eGFP and pGPD‐mCherry to measure the fluorescence values at different times up to 36 h. Expression of mCherry and eGFP under the control of the GPD1 promoter was confirmed by fluorescence microscopy (Figure S2). The results showed a linear increase in mCherry and eGFP fluorescence with OD in our strains (Figure S3). This showed the stability of mCherry in a high‐copy‐number plasmid and eGFP in a low‐copy‐number plasmid. The ratio of mCherry/eGFP gradually converged on a fixed value after the exponential growth period (Figure S3). Although these data showed that mCherry/OD could be used to screen the candidate promoters in this study, using eGFP as a reference eliminated effects of some other influencing factors and improved the reliability of the data. For example, factors that influence protein expression, such as translation (rather than transcription), could be eliminated by using the chosen eGFP as an internal reference.

**Figure 2:**
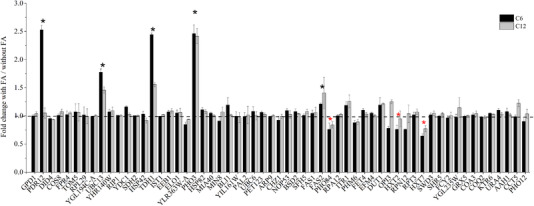
Measurements of the response of selected promoters to C6 and C12 fatty acids (1 mM). The mCherry fluorescence reporter was ligated behind the promoter of the selected gene (*x*‐axis). A strain containing plasmids pLeu2‐GPD‐mCherry and pRS316‐GPD‐eGFP was used as the control. The tested promoters were the region around 1.5 kb upstream of each candidate gene. *S. cerevisiae* strains were cultured in SD‐URA‐LEU medium with fatty acids and collected after 16 h. Fluorescence data for mCherry and eGFP were measured after diluting the cell OD to 1. Data were analyzed by the fluorescence ratio of mCherry to eGFP and fatty acids/(mCherry/eGFP without fatty acids), and data represent the mean ± SD of three biological replicates. Black asterisk‐labeled fatty acid‐responsive promoters upregulated mCherry expression, and red asterisk‐labeled promoters downregulated mCherry expression

Because we wanted to identify MCFA‐responsive promoters, we decided to separately add C6 or C12 fatty acid to culture medium and then measured fluorescence data for mCherry under the control of each promoter to be tested and eGFP under the control of the promoter of GPD1. A fresh colony was cultured directly in medium containing 1 mM fatty acid (C6 or C12) and the initial OD was adjusted to 0.05. After about 16 h, when the OD of the cells was < 3, fluorescence data for mCherry and eGFP were measured after diluting the cell OD to 1. It should be noted that when fatty acid was added to the medium, there was no obvious influence on cell growth after 16 h. As expected, the positive control with plasmids pLeu2‐GPD‐mCherry and pRS316‐GPD‐eGFP showed high expression of mCherry but no response to C6 or C12 fatty acids (Figure 2). eGFP under the control of promoter GPD1 also showed no response to C6 or C12 fatty acids (Figure S4). Most of the tested promoters showed only a small response to the fatty acids (Figure 2), and some showed no response. One promoter, of *PHO3* (an acid phosphatase), showed the opposite response compared with that in the transcriptomic data. Thus, there were some inconsistency between the screened promoters and the transcriptomic data; however, tests of some promoters also contradicted RNA‐Seq results in previously published articles 4, 35. It has been suggested that transcriptional change is sometimes transient and may not cause an increase in protein expression 35.

Among the screened promoters, we identified the previously reported short‐ and medium‐chain fatty acid promoter sensor pPDR12 28. We also identified three further promoters—pTDH1, pPHO3, and pUBC13—that obviously upregulated mCherry expression upon exposure of cells to C6 or C12 fatty acid. pTDH1 was more sensitive to C6 than to C12. The promoter pFAS2 that apparently upregulated mCherry expression was less sensitive to C6 or C12 than pTDH1, pPHO3, and pUBC13 (Figure 2). Other promoters showed less sensitive responses than those above. We also identified two promoters—pHXT7 and pPHO84—that obviously downregulated mCherry expression when cells were exposed to C6 or C12 fatty acid. pPHM6 slightly downregulated mCherry expression upon C6 and C12 exposure, and pHXT2 downregulated mCherry expression but only in response to C6 (Figure 2).

In the experiments described above, mCherry/eGFP was used for analysis with eGFP (under control of promoter GPD1) as an internal reference. We also wished to determine whether data obtained using mCherry/OD were consistent with those obtained when eGFP expression was used as a reference. The results (Figure S5) showed that promoters with high response to fatty acids could be identified by both analysis methods, but there were some differences in the results for less responsive promoters. We chose the highly responsive promoters (marked by asterisks in Figure 2) for further analysis.

Some of the promoters we identified, including pTDH1, pHXT7, and pHXT2, have been studied 46, but they have never been reported as responding to fatty acids. pTDH1 is possibly a multi‐stress‐induced promoter responding to, for example, high osmolarity 47, heat shock 48, microcultivation 49, reductive stress 50, and the stationary phase 48. The osmotic stress response of pTDH1 is related to transcription factor Msn2/4p 47, and Msn2/4p is also involved in the weak acid stress response 51. The promoters of HXT2 and HXT7 are induced by low glucose concentrations, and HXT7 was induced by weak acid stress 51. One reason that high glucose represses the promoter of HXT2 is related to transcription factor Snf1p 46. Snf1p is also a regulator of lipid accumulation in *Yarrowia lipolytica* 52, although it is not known whether there is any relationship between the response of HXT2 to fatty acids and Snf1. pUBC13, the promoter before a DNA damage‐inducible protein, is involved in cellular tolerance to DNA‐damage 53. pPHO3, a promoter before a thiamine‐repressible acid phosphatase gene, was reported to contain an activation element in the promoter region that binds a regulatory protein 54, 55. pFAS2 56 is a promoter before a fatty acid synthase subunit gene. pPHO84 57, containing a Pho4 binding site, is a promoter before a phosphate transporter gene 58. We were not able to establish why the promoters of these genes were influenced by fatty acids, and this requires further investigation.

### 3.3 Evaluation and characterization of fatty acid‐responsive promoters

#### 3.3.1 Reponse of fatty acid‐responsive promoters to different chain‐length fatty acids

To investigate the response of promoters to fatty acids with different carbon chain‐lengths, we added 1 mM even number fatty acids from C2–C16 to growing cells. Addition of fatty acid to the medium did not influence cell growth, and, after 16‐h cultivation, OD of the cells reached about 2.5 in each case (data not shown). Then, fluorescence data were measured after diluting the cell culture OD to 1.

**Figure 3:**
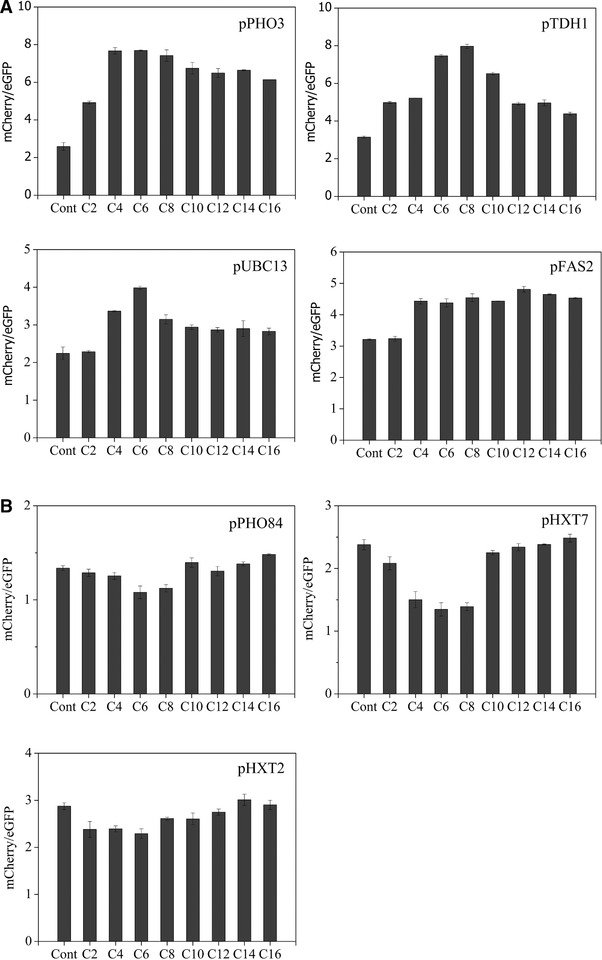
Measurement of the response of selected promoters to different carbon chain‐length fatty acids (1 mM). *S. cerevisiae* strains were cultured in SD‐URA‐LEU medium and collected 16 h after the addition of fatty acid. Fluorescence data for mCherry and eGFP were measured after diluting the cell OD to 1. Data were analyzed by mCherry/eGFP fluorescence ratio and represent the mean ± SD of three biological replicates. “Cont” indicates that the strains containing the selected promoters were cultured in the same medium but without fatty acids. Promoters could upregulate gene expression upon exposure to fatty acids (A), or downregulate gene expression (B)

**Figure 4:**
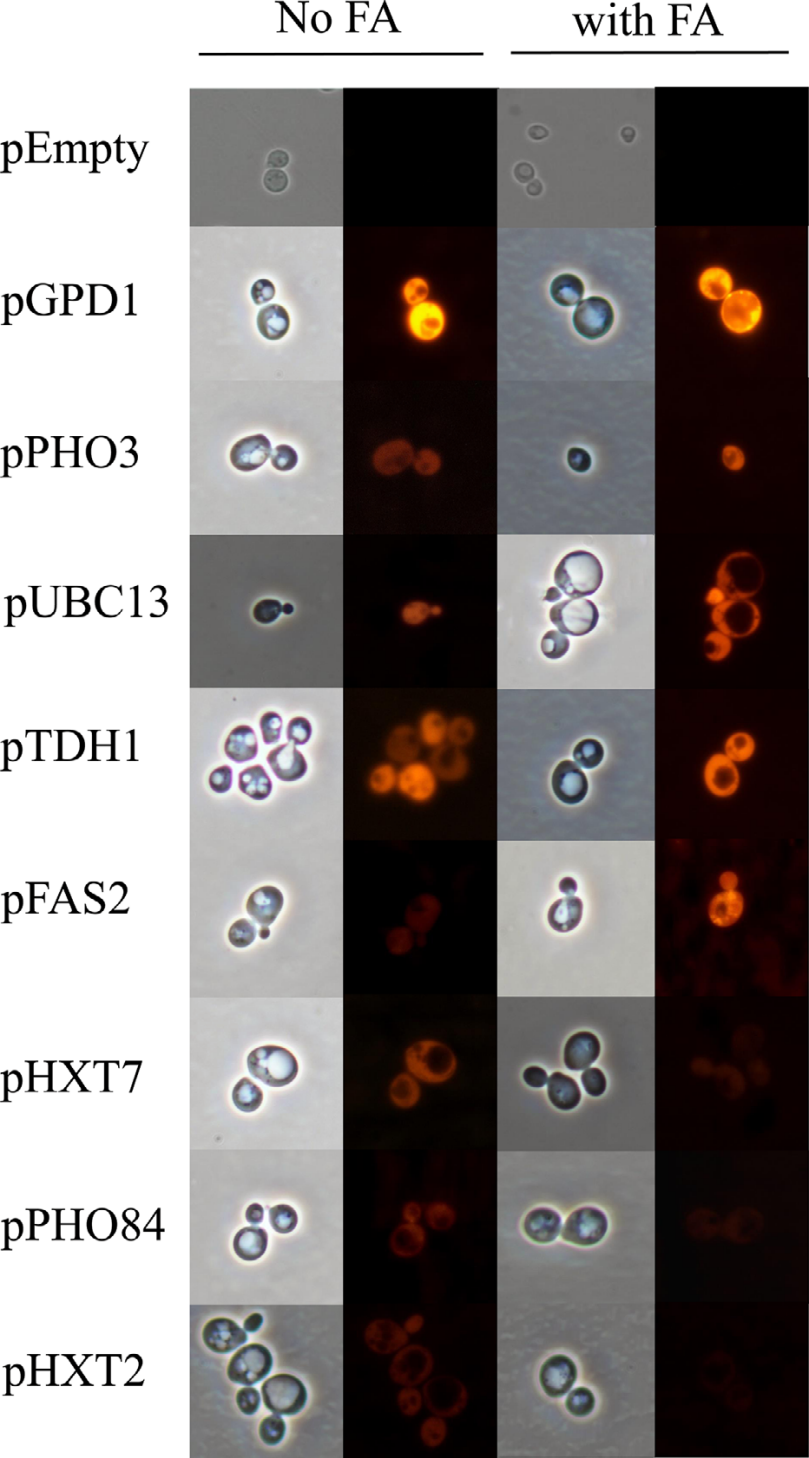
Verification of selected promoters by detecting mCherry expression by fluorescence microscopy. “No FA” indicates that no fatty acid was added to the medium, and “FA” indicates that fatty acid was added to the medium. The fatty acid in Figure 3 to which the promoter was most sensitive was added to 1 mM in the initial culture stage for each respective promoter, and, after about 16 h, the image was observed under a fluorescence microscope. pEmpty indicates the negative control strain containing plasmid pLeu2‐Empty‐mCherry

The control strain containing mCherry under promoter GPD1 showed no response to the fatty acids added to the medium. eGFP under the control of promoter GPD1 also showed no response to the added fatty acids (Figure S4). We found that the selected promoters from the upregulation group responded to MCFAs but with different characteristics (Figure 3A). pPHO3 and pUBC13 were more sensitive to C4–C8 fatty acids, and pTDH1 to C6–C10 than to other carbon chain‐length fatty acids. Although pPHO3 and pUBC13 were more sensitive to short‐ and medium‐chain fatty acids, they were also responsive to longer‐chain fatty acids (C8–C16) with pPHO3 showing the greater sensitivity. pTDH1 showed little response to C16, consistent with the initial screening result in which this promoter showed its greatest sensitivity to C6 (Figures 2 and 3). pFAS2 showed a broad response to C6–C16 but with less sensitivity compared with the other identified promoters that upregulated gene expression; pFAS2 was most sensitive to C12 fatty acid. In the downregulation group, the tested promoters did not significantly respond to the added fatty acids. pPHO84 showed a slight response to C6 and C8. pHXT7 was more sensitive to C4–C8 fatty acids than to other fatty acids. Compared with pHXT7, pHXT2 showed less response to fatty acids from C2 to C12 (Figure 3B). We verified each selected promoter controlling the expression of mCherry by using a fluorescence microscope with cells grown in SD medium with and without the addition of fatty acid, and found that all the promoters we selected were functional (Figure 4).

We also tried to identify the characteristics of seven promoters when cells were cultured in a minimal medium. Strains containing the selected promoters controlling expression of mCherry with eGFP under the control of GPD1 cultured to OD ∼2.5 were used to measure fluorescence data. In minimal medium, the promoters could respond to the added fatty acids, but with some decreased fold‐changes compared with growth in SD medium. We speculate that this was because of the different growth conditions (Figure S6). The data showed some small differences between the initial transcriptomic screening and responses to fatty acids in culture experiments, and we assumed that the differences were due to the growth conditions and the sensitivity of the promoters (Figures 2 and 3).

#### 3.3.2 Dose–response of the fatty acid‐responsive promoters

To determine the dose–response of promoters to the fatty acid(s) to which they were most sensitive, we chose pTDH1 and pPHO3, which showed high responses to short‐ and medium‐chain fatty acids, and pFAS2, which was responsive to MCFAs. These promoters respond to fatty acids and then upregulate gene regulation. Moreover, two promoters, pHXT7 and pHXT2, that respond to fatty acids and downregulate gene expression, were also chosen to test their dose–response to the fatty acid to which they were most sensitive. Because of the solubility of fatty acids, we set the maximum fatty acid test concentration to 1 mM.

**Figure 5:**
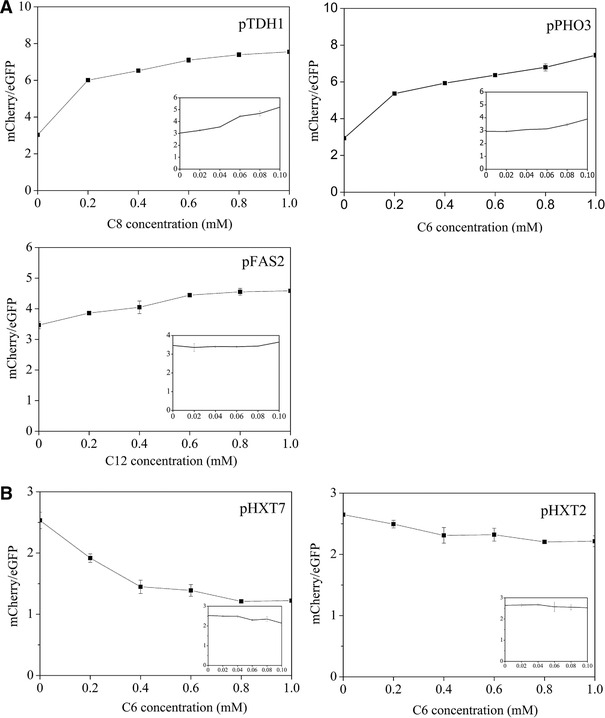
Dose–response tests of the selected promoters to different concentrations of fatty acids. The fatty acid selected for each promoter was the one to which the respective promoter was most sensitive (Figure 3). The inset figure shows the response to low concentrations of fatty acid (0.02–0.1 mM). *S. cerevisiae* strains were cultured in SC‐URA‐LEU medium and collected 16 h after the addition of fatty acid. Fluorescence data for mCherry and eGFP were measured after diluting the cell OD to 1. Data represent the mean ± SD of three biological replicates. Promoters could upregulate gene expression upon exposure to fatty acids (A), or downregulate gene expression (B)

We found that, except pFAS2 and pHXT2, these promoters could respond well to just 0.2 mM fatty acid (Figure 5). pFAS2 and pHXT2 also responded to 0.2 mM fatty acid, but with less sensitivity than the other tested promoters. Moreover, except pFAS2 and pHXT2, all the promoters showed some response to fatty acids at < 0.1 mM. pTDH1 and pPHO3 both showed high response in our experiments to short‐ and medium‐chain fatty acids, but pTDH1 was more sensitive than pPHO3 because pTDH1 responded even on exposure to just 0.02 mM fatty acid, and showed high activity when the fatty acid concentration reached 0.06 mM. pHXT7, belonging to the downregulation group, could respond to 0.06 mM C6 fatty acid (Figure 5).

The promoters identified in this article could respond to MCFAs. Some of the screened promoters showed high sensitivity. High sensitivity toward low concentration products is sometimes necessary for dynamic regulation using promoters. The promoters we screened, such as pPHO3, can also respond to LCFA but pPHO3 responds more sensitively to MCFAs, and the fatty acids synthesis process is from short‐chain to medium‐chain, then to long‐chain and very‐long‐chain fatty acids. So, pPHO3 can be used in the dynamic regulation of MCFAs or derived products. To further test our screened promoters and to verify the methods available to determine MCFA‐responsive promoters, we then removed the reference eGFP plasmid. Analysis of the response of promoters pPHO3, pTDH1, and pHXT7 controlling mCherry expression to different carbon chain‐length fatty acids was carried out, and the dose–response of these promoters to the fatty acids was determined using the mCherry/OD method. Similar results were obtained to those obtained with the mCherry/eGFP analysis method (Figures S7 and S8). Finally, pPHO3, pTDH1, and pHXT7 were individually placed in a single‐copy plasmid. The results showed that the three promoters worked in this plasmid, but with less sensitivity compared with their response when in a multicopy plasmid (Figure S9).

In our next work, we will use some upregulating responsive promoters to improve MCFA synthesis, and then use downregulating responsive promoters to turn down long‐chain and very‐long‐chain fatty acid synthesis. The upregulating responsive promoters will also be used in the heterologous medium‐chain dicarboxylic acid pathway we constructed previously 43, to analyze the dynamic regulation effect.

## 4 CONCLUDING REMARKS

We screened for promoters in *S. cerevisiae* that respond to different carbon chain‐length fatty acids, especially to medium‐chain fatty acids. The promoters we identified include examples that up‐ and downregulate gene expression. In our next work, we will try to apply them in dynamic regulation to improve the production of MCFA‐derived chemicals in *S. cerevisiae*.

## CONFLICT OF INTEREST

The authors have declared no conflict of interest.

## Supporting information

Supporting Information
