# Supplementary material for: Discovery and identification of medium‐chain fatty acid responsive promoters in Saccharomyces cerevisiae
Source: Eng Life Sci. 2020 Jan 21;20(5-6):186–96. doi: 10.1002/elsc.201900093 (PMC7447867; doi:10.1002/elsc.201900093)

Table 1. Primers used in this study

Primer number	Primers sequence
eGFP	F: TGCCACCATACCCACGCCGAAAC
	R: CCCAAGCTTTTACTTGTACAGCTCGTCCATGC
YPL058C	F: CGAGCTCAAAAGTGATGTTTACGATTTAGAA
	R: CGGGATCCTTTTTTATTAATAAGAACAATAACA
YKL002W	F: CGAGCTCGTATACTACAGCTTTTTCAA
	R: CGGGATCCTGTTTAACGTTCTGTGTCCAA
YHL048w	F: CGAGCTCGCTTAGTCGAGGAGAACCAG
	R: CGGGATCCTTTCGGTAGTGAGATGGCAGT
YLR449W	F: CGAGCTCCTCTCTAGTTCCAGGCACTGT
	R: CGGGATCCGGTAGAGACGTATATTTTG
YPR133W-A	F: CGAGCTCCTCTTGCGTTTGTAGGTACT
	R: CGGGATCCTTTATACTATCTAGTTATTT
YFR032C-A	F：CGAGCTCAAAGTGGCCCACACCGGGTTT
	R：CGGGATCCTTTCTGGAGAAGACGATGA
YGL194C-A	F：CGAGCTCCGTGAATTTTCTCTCGAAGGTT
	R：CGGGATCCAATTGTCCAGATGTATTACA
YDR092W	F：CGAGCTCCGATTTTTGAAGTTGGAGTG
	R：CGGGATCCTTCTAACTATGTTACAATG
YHR140W	F：CGAGCTCAGGTCCACCCGTGATGAT
	R: CGGGATCCGGATTCTCCTTGTATTATGGT
YEL024W	F: CGAGCTCAAAGAAATAGTATCCAGTT
	R: CGGGATCCGTTTGTTATTGCTCCTTTCG
YPL087W	F：CGAGCTCTTAAGCAGGTAAGACTCGGT
	R：CGGGATCCTTGATTTTTTTCTTTATCTTAC
YLL041C	F：CGAGCTCCCCTATAAATATGTATACTCCAG
	R：CGGGATCCGCGGGTGATATATATTTCGA
YDR171W	F：CGAGCTCCGGCAAAAGCACCAAACACAG
	R：CGGGATCCTGCTTCGGCTTGGTATGATCTT
YJL052w	F：CGAGCTCTTAAGCTTGATCACAAGAGA
	R：CGGGATCCTTTGTTTTGTGTGTAAATTTAGTGA
YNL265C	F: CGAGCTCCTTTTCTTATTTTTCCTTTC (低 GC)
	R：CGGGATCCTTTAGTTGTCGTATTCTCTTA
YPL095C	F：CGAGCTCAAACGCTGCAACTCCTAAAT
	R：CGGGATCCTAAAATGACAAATATGAAAA
YJL196C	F：CGAGCTCGTTGGAAGATAATAGTGGGC
	R：CGGGATCCACTTGCTTGCAAATTTAG
YLR363W-A	F：CGAGCTCGGCCTAGTGCCCTTGAATTG
	R：CGGGATCCATTGTAGCTTATGGACTTTATGAA
YBR092C	F：CGAGCTCTCCGTGATGACGATGATTTG
	R：CGGGATCCAGGTAATTTGGAATGGCCCT
YPL240C	F：CGAGCTCAGAAAACAAGCTCTTTACTTTAGTC

	R：CGGGATCCATCTTTGCGTGTTTGTTTGTGCT
YKL195W	F：CGAGCTCGAGAGCACTCATAGAGAGTA
	R：CGGGATCCTTTACTGATAATATACACTC
YPR010C-A	F：CGAGCTCCCCTTCAATATTTTTTTAC
	R：CGGGATCCTTTTCTATATTTTTCTTT
YKL061W	F：CGAGCTCATTTTTCTAAGGACTCTGG
	R：CGGGATCCTTCCCTACTCCTTTTAGTAC
YIL161W	F：CGAGCTCGGATTATGACGGCTGCCA
	R：GGACTAGTTTCCCTCACTACTTCTTT
YHR097C	F：CGAGCTCCTATTAACACGTCAGGTGCGG
	R：CGGGATCCTTGCGTCTGTTTGATATAGT
YER100W	F：CGAGCTCTCTGTTAGTACATTTATACA
	R：CGGGATCCTACTATTGTACGTACTTTG
YOR158W	F：CGAGCTCTTAATTTTAGTGTTATG
	R：CGGGATCCACTTCAATTATCCTTGTTAT
YMR033W	F: CGAGCTCATATGAGAAACCATTGCC
	R: CGGGATCCATTCAATGCATATAGTAGT
YER030W	F：CGAGCTCGAAGCACACCGACTCATGGC
	R：CGGGATCCTATTTCTCTCTTTCCTTCC
YPL146C	F: CGAGCTCTCGACAGGTCTACTTTGTG
	R: CGGGATCCTTTTAAATAAATGATTCA
YBR290W	F：CGAGCTCACACTTTGTTATGGAACAA
	R：CGGGATCCAGCGCTTAGTTTCCTAGACT
YJL145W	F：CGAGCTCCAAGCGAAAGGGCATCACCA
	R：CGGGATCCTTTAAGGATGTTAATTTAGTG
YKL182W	F：CGAGCTCCGACGGCGAAGAAGCACAT
	R：CGGGATCCAATGAGCGAATAATAGAG
YPL231W	F：CGAGCTCAGAAAGGTCTATCTCACG
	R：CGGGATCCAATGATGGTATTTCAATTGGTG
YML123C	F：CGAGCTCTTTTGGCGTCGGGAGAGTAC
	R：CGGGATCCTTGGATTGTATTCGTGGAGT
YJR063W	F：CGAGCTCATTCTTTCCTTGGAACTTC
	R：CGGGATCCTCTTATAAGTACTAACCTGG
YDR497C	F：CGAGCTCGCAAGATGCATTATTAAAT
	R：CGGGATCCTTTAACACCCACTGCAGAA
YDR281C	F：CGAGCTCCAGAACTAATTGAGACCCC
	R：CGGGATCCCTTGGTGTCAGGTTGAAAA
YMR319C	F: CGAGCTCCACTAAATGAAAACATCTGC
	R：CGGGATCCAATTTGATTAACGAATGTT
YIL064W	F: CGAGCTCCACTAAATGAAAACATCTGC
	R：CGGGATCCAATTTGATTAACGAATGTT
YBR252W	F：CGAGCTCCAAATGGAAAAGTTTTACAA
	R：CGGGATCCTTGGTTATTTTTTGGCTCGC
YJR073C	F：CGAGCTCGCCCTTTATATCTTGAACACG

	R：CGGGATCCCGCTTGACTTGCGCTATTC
YMR011W	F：CGAGCTCCTAAACAAAATGTGCCTGAC
	R：CGGGATCCTATGTTGCTTTATAAGTCTT
YFR052W	F：CGAGCTCTGCGGCACCCGCTCTTCTT
	R：CGGGATCCTACGATGGCTTCTTATCTCACT
YDR394W	F：CGAGCTCCAAGGCCAAAAAATCAAT
	R：CGGGATCCTCTTGTTGTGGTTTTTACTTTA
YDR342C	F：CGAGCTCATGGGGGATTAAATCCGG
	R：CGGGATCCTTTTTGATTAAAATTAAA
YBR175W	F：CGAGCTCCACTCCAAGATTAGCAGCAT
	R：CGGGATCCGTTAGCATATAATGGCCACT
YOL110W	F：CGAGCTCCTCTGCAGGCTGCCTACGCT
	R：CGGGATCCCTAGTAAGGGTTAGATTGAG
YER056C	F：CGAGCTCACGCCACTGTTTCCAAGACC
	R：CGGGATCCTCTTATAAATCGTTATAATAG
YGL235W	F：CGAGCTCGACTGGTTGGCTTGGCTTCCG
	R：CGGGATCCCAAAGGTGTGGTTCTGGATGAC
YPL059W	F：CGAGCTCTTACTCTCGTTCCAACAATC
	R：CGGGATCCTGTCTGATTTCCTCAAATT
YJL062W-A	F：CGAGCTCGTTGTTGGTATCACATCATT
	R：CGGGATCCACTACTTTGTCGTCTATATATGT
YNR041C	F：CGAGCTCGTAGGGAAACGTAAATTAGCT
	R：CGGGATCCTAGTTGACACTGTTATTCT
YPL053C	F：CGAGCTCTTTATTCTCCTTCATCTACT
	R：CGGGATCCCTTACCTTATCTTATCTTCACT
YLR420W	F：CGAGCTCCCAAGTTCAGTCTTATTTTCAAC
	R：CGGGATCCTGCTTTGTTAGGTATTACTT
YNL141W	F：CGAGCTCCGTAAAGGGATGCCCAAGTAC
	R：CGGGATCCTATTTTTTTTTTCTTTTC
YJR064W	F：CGAGCTCTAGAGAAAAGTCTTTCTGTA
	R：CGGGATCCTTTCAAATATTGCTATAAAAATGG
YHR215W	F：CGAGCTCGAACTCAGCTAAGACTCGAC
	R：CGGGATCCTGGTATTTCTGATGATGTTC

Table 2. The data from the heat maps presented in Figure 1A and the data of selected promoters responded to fatty acid C6 and C12. Values are log 2 ratios (FAs/No FAs) of transcriptome analysis (fatty acids of C6， C12 and C16) and fluorescent output from various promoters (sensor), measured after 16 hours, in cells harboring pRS316-GPD-eGFP and pLeu2-promoter-mcherry and culture broth containing 1 mM C6 or 1 mM C12.

Gene	C6	C12	C16	Cont（FPKM）	C6（FPKM）	C12（FPKM）	C16（FPKM）		Sensor output responsive to C6, ratio of (mcherry/GFP with C6)/(mcherry/GFP without C6)	Sensor output responsive to C12, ratio of (mcherry/GFP with C12)/(mcherry/GFP without C12)
PDR12	15.64	0.00	0.00	0.00	511.50	0.00	0.00		2.53	1.05
DID4	12.18	0.00	0.00	0.00	46.36	0.00	0.00		0.95	0.93
COS8	5.41	-7.19	-7.19	1.46	62.17	0.00	0.00		1.01	1.08
FPR4	11.03	11.72	11.49	0.00	20.85	33.79	28.76		1.02	1.06
TOM5	0.00	12.80	0.00	0.00	0.00	71.25	0.00		1.07	1.06
RPL29	0.00	12.88	0.00	0.00	0.00	75.39	0.00		1.02	0.99
YGL194C-A	3.60	1.30	2.68	3.23	39.12	7.93	20.74		0.99	1.01
UBC13	2.88	3.53	2.34	4.35	31.92	50.20	22.07		1.77	1.46
YHR140W	2.48	3.82	0.99	9.71	54.25	137.55	19.29		1.07	1.09
RIP1	2.44	0.86	0.58	19.17	104.34	34.90	28.70		1.01	1.01
YDC1	2.09	0.83	0.64	22.98	98.02	40.72	35.87		1.16	1.02
SDH2	2.56	0.50	0.68	22.67	133.58	32.16	36.38		1.01	1.01
HSP42	3.47	0.58	-0.08	7.99	88.52	11.95	7.57		1.03	0.92
TDH1	3.16	-0.07	0.40	440.05	3927.72	418.88	579.30		2.45	1.56
IST1	2.61	-0.10	-0.55	7.96	48.73	7.41	5.43		0.99	1.02
EEB1	0.35	3.73	0.12	22.91	29.17	303.19	24.86		1.07	1.09
ELO1	-0.23	2.00	0.80	211.23	180.00	846.27	367.87		1.05	1.06
YLR363W-A	-2.16	-2.13	-0.79	56.17	12.56	12.87	32.49		0.85	0.94
PHO3	-0.52	-3.03	-0.20	42.82	29.87	5.23	37.36		2.46	2.42
HSP82	1.29	-4.48	-0.65	32.94	80.72	1.48	21.01		1.11	1.08
MIA40	1.97	-2.94	-0.21	18.29	71.78	2.39	15.76		1.05	1.00
MIN8	1.63	-2.23	-0.37	29.08	90.00	6.18	22.49		0.91	1.07
BLI1	1.16	-0.95	-1.27	20.26	44.24	10.44	8.37		1.19	1.01
YIL161W	1.16	-1.96	-0.65	17.89	39.88	4.59	11.43		0.99	0.97
PAL2	0.83	-1.86	-1.07	19.80	32.04	4.96	8.55		0.99	1.02
UBC6	0.68	-1.66	-0.78	42.55	68.10	13.48	24.83		1.08	1.00
PET123	1.21	-1.16	0.23	27.26	62.88	12.18	31.88		1.06	1.02
ARP9	1.08	-0.99	-0.49	20.63	43.72	10.41	14.70		0.99	1.01
CHZ1	0.84	-1.54	-0.17	132.87	237.83	45.64	118.51		0.92	0.99
NOP53	0.42	-1.62	-0.04	49.92	66.56	16.21	48.48		1.09	1.03
BSD2	0.89	-1.99	-0.09	20.76	38.38	5.24	19.51		1.08	1.04
SFH5	0.37	-2.12	0.08	34.89	45.23	8.04	36.85		1.01	1.08
FAS1	-1.81	1.00	-1.02	40.40	11.50	80.55	19.95		1.05	1.06
FAS2	-1.60	0.54	-0.70	74.38	24.48	108.04	45.74		1.21	1.41
PHO84	-1.77	0.26	-0.36	171.78	50.46	205.38	134.11		0.76	0.84
RPA12	-1.92	0.97	0.85	44.34	11.71	87.14	79.85		1.00	1.03
ITR1	-1.99	0.34	0.08	536.58	134.97	678.43	567.38		1.19	1.26
PHM6	-2.10	0.60	0.30	78.79	18.35	119.79	97.02		0.88	0.89
FET4	-2.50	0.13	0.31	65.71	11.64	71.90	81.46		1.10	1.03
EFM4	-3.01	1.05	0.21	37.96	4.72	78.58	43.80		1.05	1.00
DUT1	-3.16	0.16	0.06	114.43	12.76	127.83	119.65		1.19	1.21
OPI3	-4.07	0.41	0.20	1503.71	89.28	1991.36	1731.36		0.78	1.25
HXT2	-6.62	0.06	-0.35	118.93	1.21	124.30	93.14		0.76	0.95
RPN12	-6.67	-12.03	-0.83	41.74	0.41	0.00	23.53		0.76	1.04
RPT3	-9.94	1.66	-9.94	9.82	0.00	31.01	0.00		1.02	1.07
HXT7	-10.70	3.09	1.45	16.62	0.00	141.28	45.50		0.64	0.77
SWD3	-10.43	1.66	1.61	13.76	0.00	43.58	41.90		1.02	1.04
SHR5	-11.33	0.61	1.67	25.71	0.00	39.11	81.98		1.00	1.04
FCY2	-13.42	0.54	-0.26	109.76	0.00	159.58	91.75		0.96	1.01
YGL235W	-13.55	0.01	0.18	24.85	9.15	40.45	34.12		0.97	1.15
GRX5	-13.55	0.01	0.18	120.29	0.00	121.18	136.03		1.00	1.01
COA3	-11.75	1.01	0.33	34.45	0.00	69.45	43.23		1.02	1.04
COQ2	-12.02	0.93	0.36	41.39	0.00	78.81	53.19		0.98	0.98
KTR6	-12.12	-0.18	-0.09	44.59	0.00	39.25	41.95		1.05	1.03
URA4	-12.43	0.21	0.05	55.30	0.00	64.10	57.27		1.10	1.03
AAH1	-12.69	-0.19	-0.54	66.06	0.00	58.04	45.40		1.08	1.02
CCT5	-11.88	0.34	-0.66	37.65	0.00	47.76	23.79		0.99	1.23
PHO12	-11.72	0.11	-0.76	33.77	0.00	36.45	19.95		0.90	1.04

Figure 1. Transcriptome analysis of *S. cerevisiae*'s response to different carbon chain fatty acids. The yeast cells were treated for about 6 h with the relevant chemical and subjected to RNA-seq analysis. (**A**) Venn diagrams showing the number of genes that were significantly up- or down-regulated upon exposure to one of the three fatty acids. (**B**) Gene Ontology (GO) terms analysis for the expressed genes in common of different fatty acids. (**C**) Gene Ontology (GO) terms analysis for the differentially expressed genes of different fatty acids.

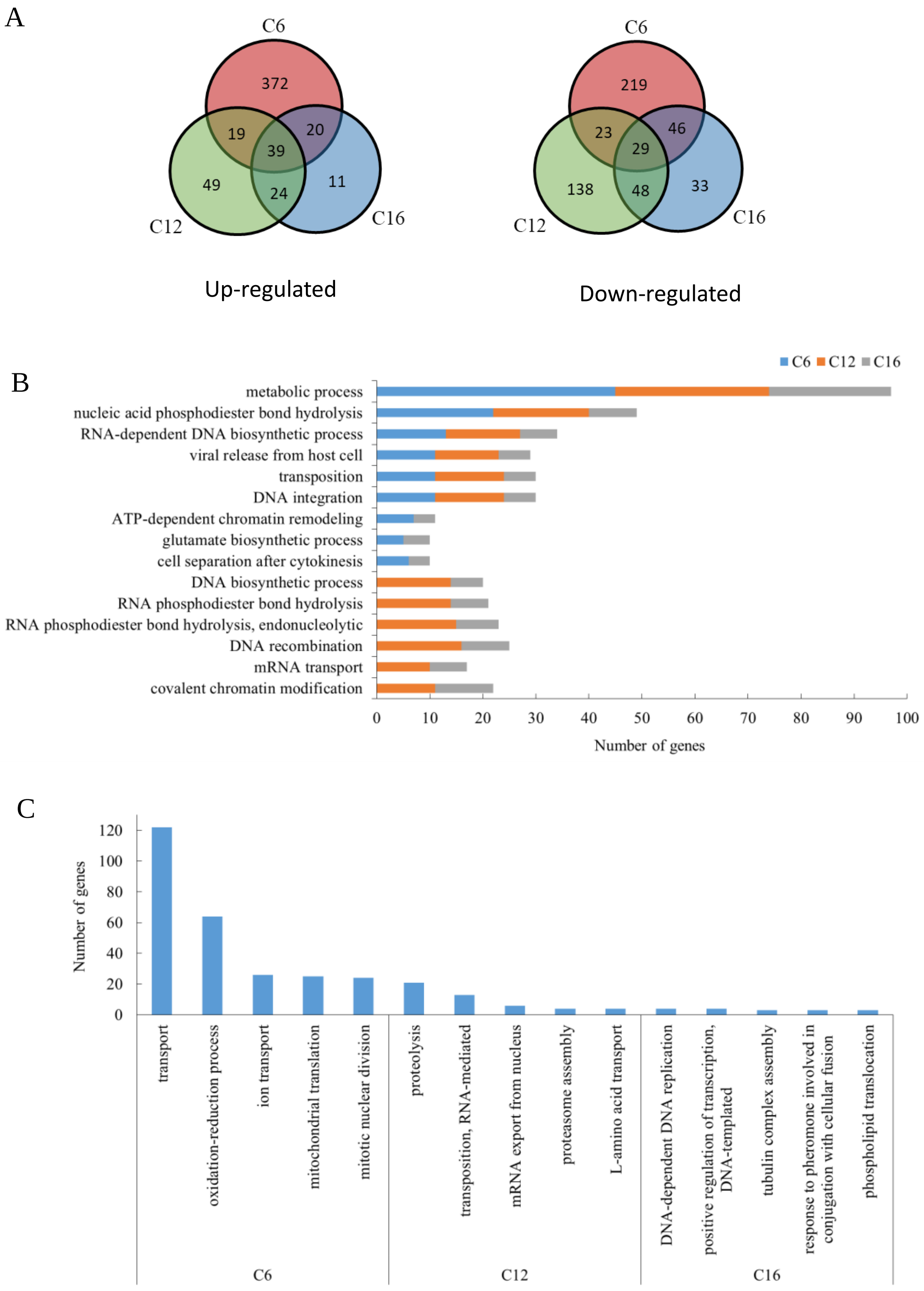

Figure 2. Verification of mCherry and eGFP expression by using fluorescence phase contrast microscopy. The upper panel displayed the strains transformed with plasmids pRS316-GPD-eGFP and pLeu2-GPD-mCherry. And the lower panel displayed the control strain transformed with plasmid pRS316.

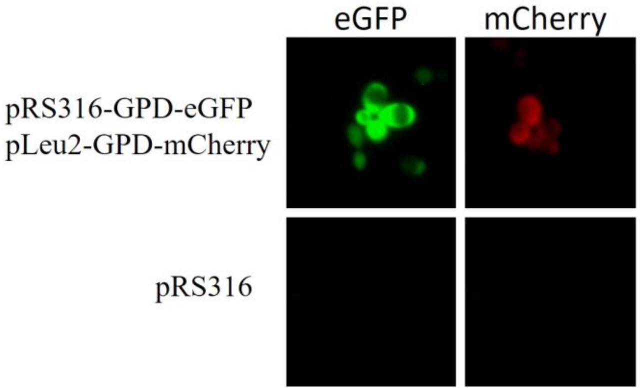

Figure 3. Verification of the viability of the promoter screening method with mCherry/eGFP. 24 samples of strains from different fresh colonies which carried plasmids pRS316-GPD-eGFP and pGPD-mCherry in 5 mL liquid medium in the test tube to measure the fluorescence value of different times until 36h.

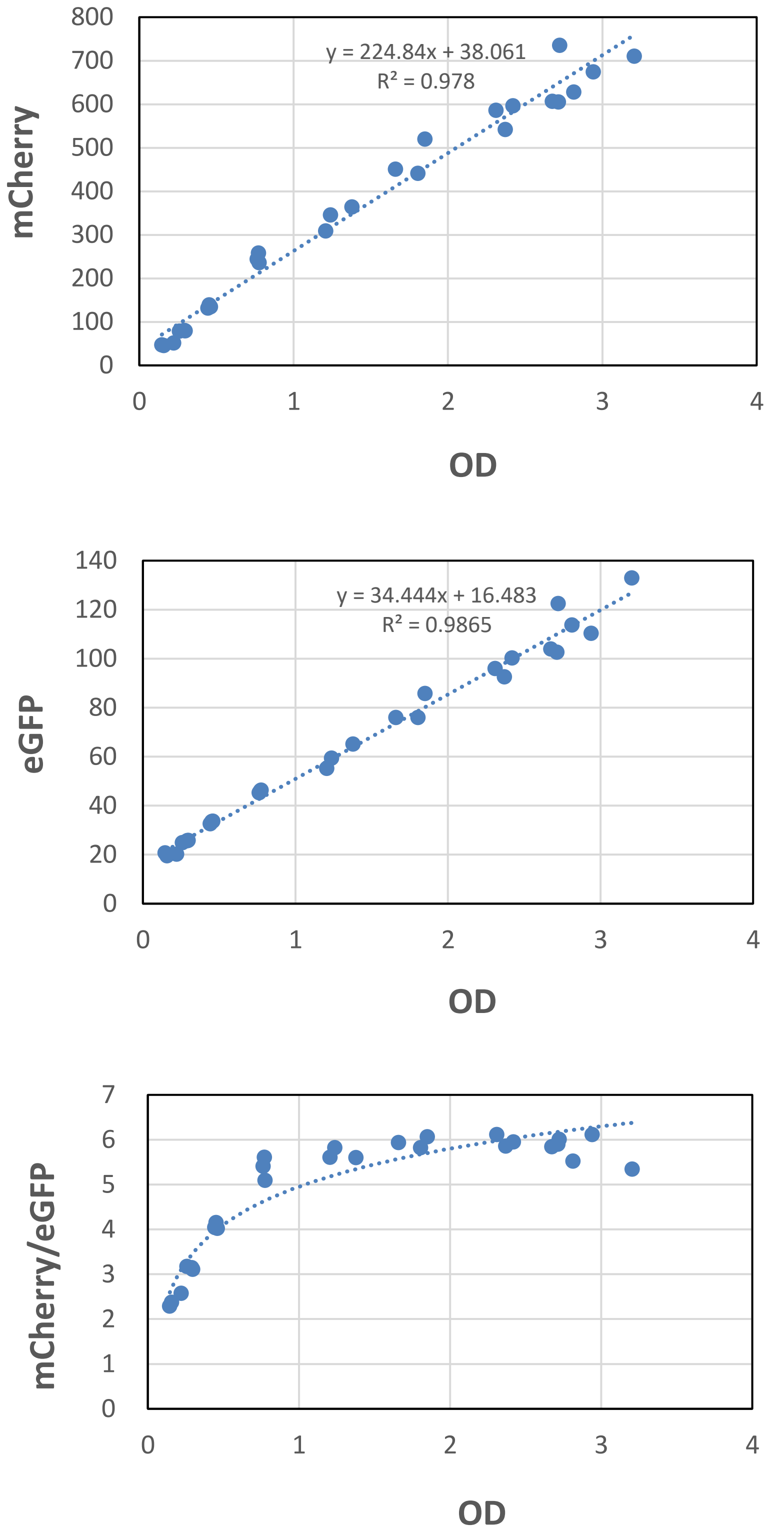

Figure 4. Detection the response of eGFP under the promoter of GPD1 to the different fatty acids (1 mM) in the engineered S. cerevisiae. *S. cerevisiae* strains containing plasmids pRS316-GPD-eGFP and pLeu2-GPD-mCherry were cultured in SD-URA-LEU medium and collected after 16 h of the initial addition of fatty acid. Fluorescence data for eGFP was measured after diluting the cell OD to 1. Data was analyzed by fluorescence fold change of eGFP with fatty acids / (eGFP without fatty acids) and data represent mean ± s.d. of 3 biological replicates.

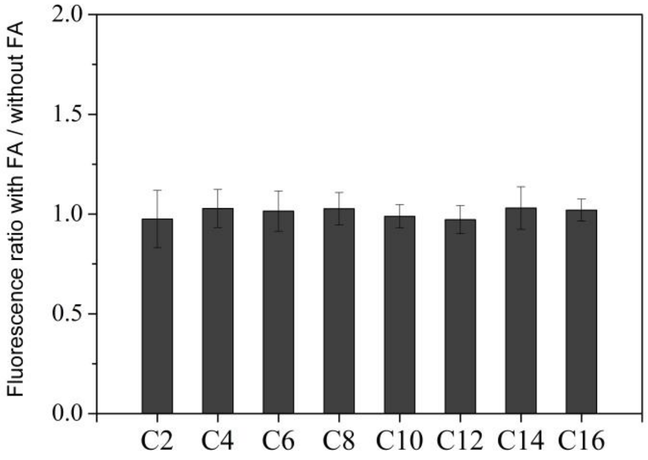

Figure 5. Measurements of the selected promoters response to fatty acid C6 and C12 (1 mM). Data was analyzed by fluorescence ratio of mcherry/OD with fatty acids / (mcherry/OD without fatty acids) and data represent mean ± s.d. of 3 biological replicates. Black asterisk labeled fatty acid responsive promoters which up regulated mcherry expression and red asterisk labeled promoters which down regulated mcherry expression.

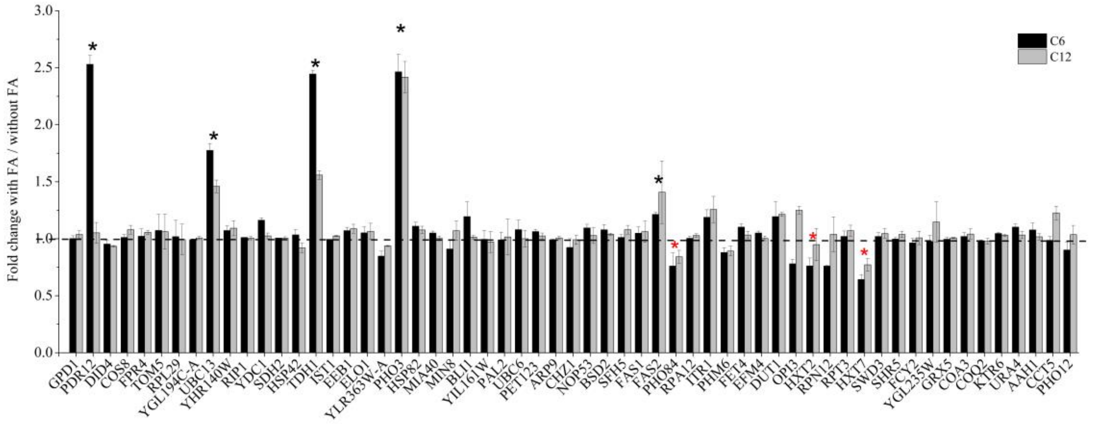

Figure 6. Verification of the seven screened promoters response to the fatty acids (1 mM) in minimal medium. Each promoter was chosen the most influential fatty acids to test the response condition. *S. cerevisiae* strains were cultured in miminal medium and collected after 16 h of the initial addition of fatty acid. Fluorescence data for mCherry and eGFP was measured after diluting the cell OD to 1. Data was analyzed by fluorescence fold change of mcherry/eGFP with fatty acids / (mcherry/eGFP without fatty acids) and data represent mean ± s.d. of 3 biological replicates. Fatty acid C6 for pGPD1, pHXT2, pHXT7, pPHO3, pUBC13, pPHO84, C8 for pTDH1 and C12 for pFAS2.

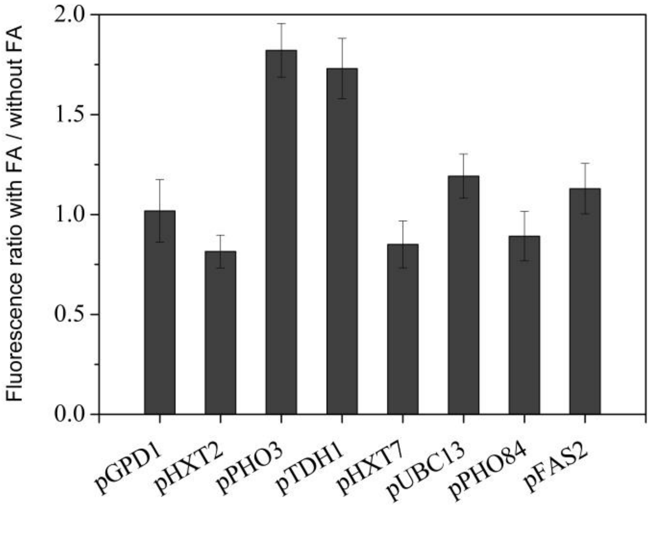

Figure 7. Measurements of the response to different carbon chain-length fatty acids (1 mM) of promoter pTDH1, pPHO3 and pHXT7. *S. cerevisiae* strains were cultured in SD-URA-LEU medium and collected after 16 h of the initial addition of fatty acid. Fluorescence data for mCherry was measured after diluting the cell OD to 1. Data was analyzed by fluorescence ratio of mcherry/OD and represented mean ± s.d. of 3 biological replicates. “Cont” displayed that the strains contained selected promoters were cultured in the same medium but without fatty acids.

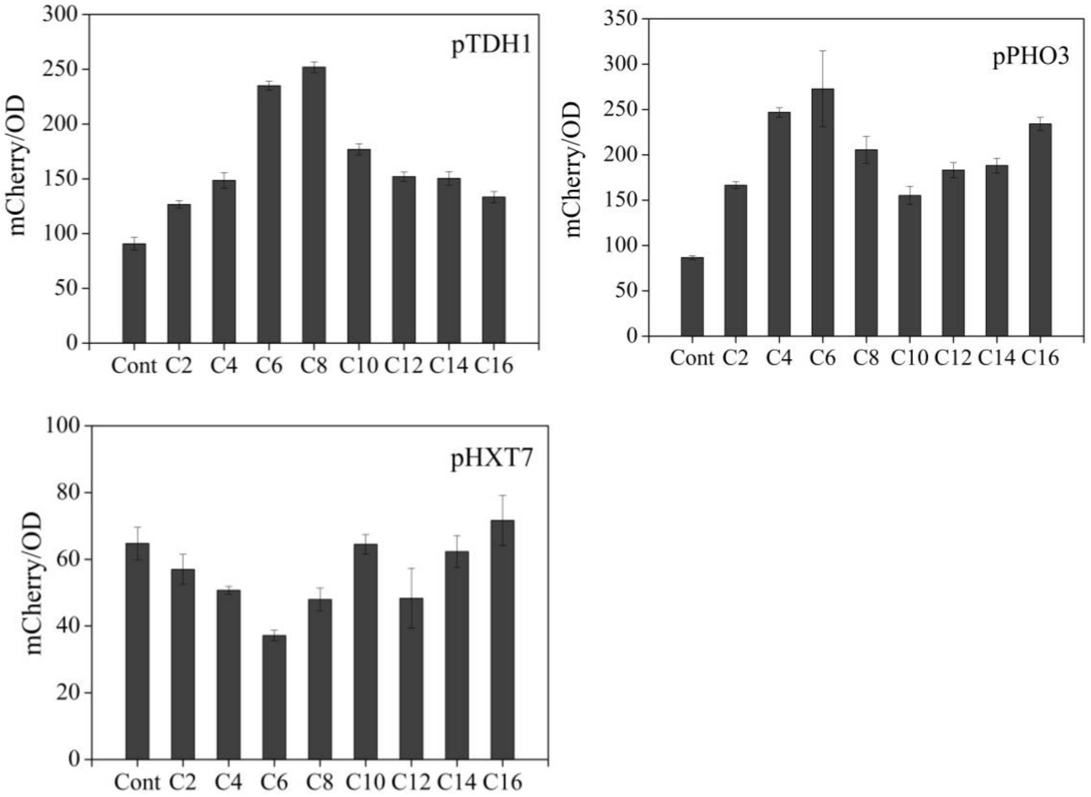

Figure 8. Dose–response tests of the selected promoters pTDH1, pPHO3 and pHXT7 to different concentration fatty acids. The fatty acid selected for each promoter was the most sensitive one in Fig. 3 in the main manuscript. *S. cerevisiae* strains were cultured in SC-URA-LEU medium and collected 16 h after the initial addition of fatty acids. Fluorescence data for mCherry was measured after diluting the cell OD to 1. Data represent mean ± s.d. of 3 biological replicates.

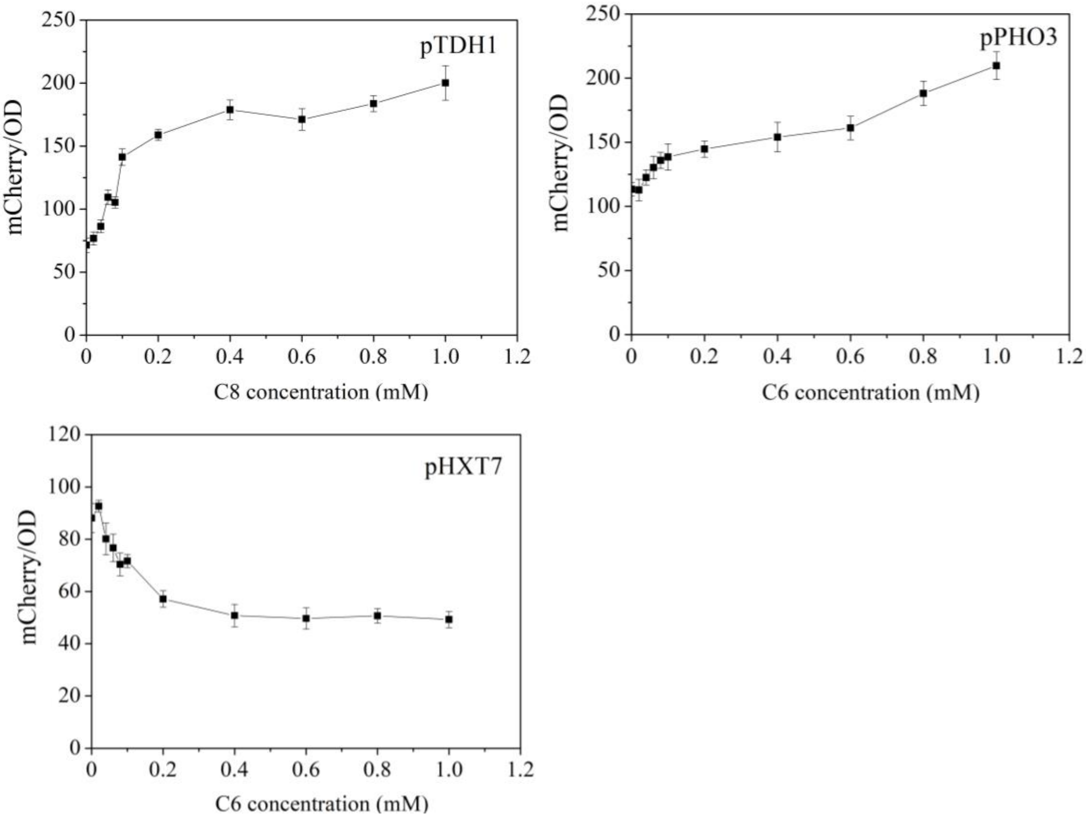

Figure 9. Measurements of promoter pTDH1, pPHO3 and pHXT7 response to fatty acid C6 in a single plasmid. The mCherry fluorescence reporter was ligated after the promoter of selected gene (x axis). The strain containing plasmid pRS316-GPD-mCherry was set to be the control. *S. cerevisiae* strains were cultured in SD-URA-LEU medium with fatty acid and collected after 16 h. Fluorescence data for mCherry was measured after diluting the cell OD to 1. Data was analyzed by fluorescence ratio of mcherry/OD with fatty acids / (mcherry/OD without fatty acids) and data represent mean ± s.d. of 3 biological replicates.

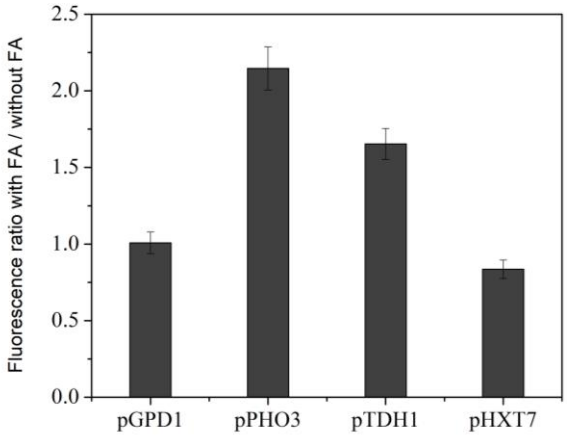

Supplement: Supplementary file 1 — Supporting Information [file ELSC-20-186-s001.pdf]
